# Tolerogenic dendritic cell-based immunotherapy

**DOI:** 10.18632/oncotarget.21867

**Published:** 2017-10-17

**Authors:** Kiyuk Chang, Jie-Young Song, Dae-Seog Lim

**Affiliations:** Dae-Seog Lim: Department of Biotechnology, Laboratory for Immune Cells, CHA University, Seongnam, Gyeonggi-do, Republic of Korea

**Keywords:** tolerogenic dendritic cells, regulatory T cells, TGF-β, inflammatory autoimmune diseases

Dendritic cells (DC) are established T-cell immunity inducers [1], and are also being viewed increasingly as T-cell tolerance mediators [2, 3]. Normally, DCs regulate immune homeostasis by maintaining the balance between T cell immunity and tolerance. Thus far, DC-based immunotherapy has been primarily a consideration in the field of cancer therapy (for the treatment of cancers using fully matured DCs). Several reports have shown that a number of obstacles must be overcome before DC-based immunotherapy can be used for general application in tumor therapy. One of the major difficulties in the treatment of advanced tumors seems to be the ability of the tumor cells to suppress the patient immune response against the tumor. It has been reported that regulatory T (Treg) cells producing TGF-β and IL-10 play a crucial role in the control of immune reactivity against self-and/or non-self antigens [4, 5]. Nevertheless, we obviously anticipate that the immunotherapy with fully matured DCs inhibits the metastasis/recurrence of cancers [6], and cytokine-induced killer cells, natural killer cells, or cytotoxic T lymphocytes directly kill established solid tumors, consequently showing respective abilities against cancers.

Among the different DC subsets, tolerogenic (t) DCs play an important role in inducing peripheral tolerance via specific mechanisms, including activation of Treg cells, suppression of effector T (Eff T) cells, and negative modulation of Th1/Th2 immune responses. Although the exact mechanisms that induce a tDC to become immunogenic or tolerogenic *in vivo* have not been elucidated, increasing evidence suggests that tDC function is pivotal for the maintenance of immune homeostasis. Recently, an intermediate stage of DC maturation was described, characterized by higher levels of MHC class II and co-stimulatory molecule expression than immature DCs (imDCs), but lacking proinflammatory cytokine secretion [2, 3]. This stage of maturation was achieved by exposing imDCs to TNF-α and a specific antigen *ex vivo*; the cells were termed antigen-specific tDCs. These tDCs induced tolerance by generating Treg cells which secreted TGF-β.

CD4^+^CD25^+^Foxp3^+^ Treg cells have emerged as a unique population of suppressor T cells, which maintain peripheral immune tolerance. Using Foxp3 as a specific molecular marker for the detection and manipulation of naturally occurring Treg, an accumulating body of evidence has shown that the CD4^+^CD25^+^Foxp3^+^ Treg population is engaged actively in the negative control of a variety of physiological and pathological immune responses, and can be exploited for the prevention or treatment of autoimmune diseases [7]. It has also been reported that TGF-β not only inhibits the proliferation of T cells, but also blocks the differentiation of both CD4^+^ and CD8^+^ naïve T cells into effector cells [7]. Moreover, TGF-β has been demonstrated to be essential for the induction and maintenance of murine and human CD4^+^CD25^+^ Treg in the periphery.

Our previous reports have further supported aforementioned logic [2, 3, 8]. Recently, Treg cells have been found to beneficially influence post-infarct healing by regulating the transition from an inflammatory phase reactive to ischemic myocardial injury to a second resolution phase in acute myocardial infarction (AMI). However, the Treg cell therapy in AMIs has some important barriers to a clinical translation, including the generation of a sufficient number of Tregs and the route of administration. As an alternative, tDCs have emerged as promising potent beneficial regulators of the post-infarct healing process via their control of Tregs and M1/M2 macrophages and have the advantage of the ease of administration and feasibility of a heart-specific tDC production. The abundance of the Treg cells in mice with an MI steered the macrophage differentiation toward the reparative M2 type, resulting in favorable left ventricular (LV) remodeling. In contrast, the depletion of Treg cells led to an impaired transition towards the resolution phase, resulting in the persistence of inflammatory M1 macrophages and delayed wound healing in the infarcted myocardium. Infarct lysate or MI mice serum-primed tDCs administered near the inguinal lymph node (LN) in MI mice migrated to regional LNs, and induced infarct tissue-specific Treg cells in the inguinal and mediastinal LNs, spleen, and infarcted myocardium indicating that a local injection of tDCs induced the systemic activation of MI-specific Treg cells, which elicited an earlier macrophage subset shift from inflammatory M1 to reparative M2 macrophages. The altered immune environment within the infarcted heart resulted in better a wound remodeling, preserved LV systolic function after myocardial tissue damage, and an improved survival.

**Figure 1:**
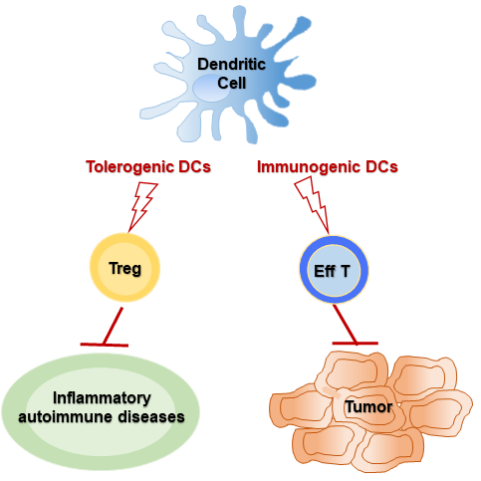
Overview of DC-mediated immunotherapy

In conclusion, our commentary provides a synopsis of the work underway that will eventually define DCs for the treatment of inflammatory, autoimmune, or cancerous diseases (immunologically different in the feature of diseases). We carefully expect that tDCs can play a crucial role in treating inflammatory/autoimmune diseases, and fully matured DCs (immunogenic DCs) can especially useful for the inhibition of cancer metastasis and recurrence rather than for the treatment of solid tumors (Figure 1).
